# Seeing an Unobservable Fe(III)/Fe(IV) Redox Process of the Nonheme Iron N4Py Complex by High-Speed Surface-Enhanced Raman Spectroelectrochemistry

**DOI:** 10.1021/acs.inorgchem.5c01017

**Published:** 2025-05-20

**Authors:** C. Maurits de Roo, W. J. Niels Klement, Daniel R. Duijnstee, Aleksandar Staykov, Wesley R. Browne

**Affiliations:** † Molecular Inorganic Chemistry, Stratingh Institute for Chemistry, Faculty of Science and Engineering, 3647 University of Groningen, Nijenborgh 3, 9747 AG Groningen, The Netherlands; ‡ International Institute for Carbon Neutral Energy Research (WPI-I_2_CNER), Kyushu University, 744 Motooka, Nishi-ku, 819-0395 Fukuoka, Japan

## Abstract

High-valent iron oxido species, central to many enzymatic and biomimetic catalyzed organic oxidative transformations, can be generated by direct electrochemical oxidation, circumventing high-energy O atom donor reagents. Electrochemical generation necessitates knowledge of the redox potentials involved, which is hindered by the lack of well-defined Fe­(III)/Fe­(IV) redox waves in the voltammetry of many iron-based catalysts. Hence, other approaches including chemical oxidation and bulk spectro­(electro)­chemical methods need to be taken. In the case of the well-studied oxidation catalyst, ${\left[\left(N_{4}Py\right)Fe\left(II\right){OH}_{2}\right]}^{2+}$ , where N_4_Py is 1,1-bis­(pyridin-2-yl)-*N*,*N*-bis­(pyridin-2-ylmethyl)­methanamine, estimates of the Fe­(III/IV) redox potentials range from 0.4 to 1.3 V vs SCE. Here, we show that electrochemical surface-enhanced Raman scattering spectroscopy reveals “hidden” redox waves, and hence redox potentials, when coupled with cyclic voltammetry. Rapid spectral acquisition (>2 Hz) of surface-enhanced Raman spectra at electrochemically roughened gold electrodes enables real-time spectral acquisition during cyclic voltammetry. We show that the Fe­(III)/Fe­(IV) redox potential of ${\left[\left(N_{4}Py\right)Fe\left(II\right){OH}_{2}\right]}^{2+}$ is close to that determined earlier by chemical redox titrations (0.85 V vs SCE). Furthermore, comproportionation and adsorption processes are shown to impact the rates of electron transfer observed, which rationalizes the absence of a distinct Fe­(III)/Fe­(IV) redox wave in its cyclic voltammetry.

## Graphical abstract

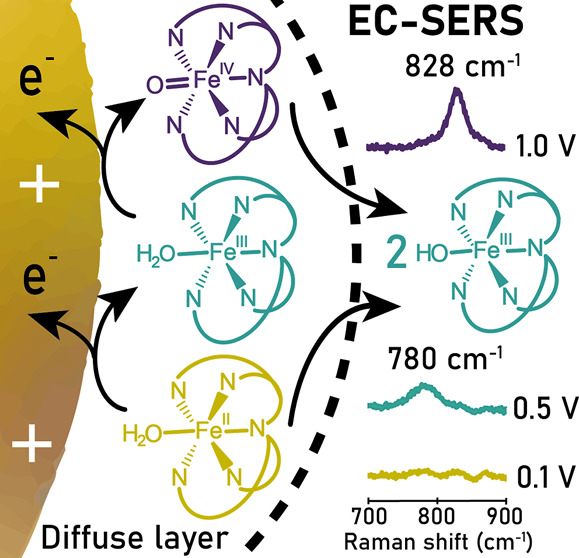

## Introduction

The design of high-valent nonheme iron catalysts for the oxidation of organic substrates is inspired by nature’s oxidation catalysts, in particular, the iron-dependent enzymes methane monooxygenase and Tau-D.^1–3^ The species that engage in substrate oxidation are typically in the Fe­(IV)=O and Fe­(V)=O redox state, and many such species have been isolated, enabling study of their reactivity under single turnover conditions.^4–7^ These species are typically generated from the Fe­(II) or Fe­(III) complex with chemical oxidants (e.g., HOCl, PhIO, peroxy acids, etc.).^5,8,9^ Recently, direct anodic oxidation has been applied to generate the Fe­(IV) and Fe­(V) oxido species with the goal to improve atom economy in the oxidation of water,^10,11^ alkene,^12^ alcohols,^13,14^ and water pollutants.^15^

Knowledge of redox potentials is essential as generation of the Fe­(IV/V)=O species has to be at less positive potentials than the direct electrochemical oxidation of organic substrates occurs at. Furthermore, these data are important in understanding the hydrogen atom (HAT) and proton-coupled electron (PCET) transfer reactions that these bioinspired iron complexes undergo.^16^

The phenyl-diamido ligand-based iron-TAML complexes of Collins and co-workers^6,17^ are exemplary, showing well-defined Fe­(III/IV) and Fe­(IV/V) redox processes at 0.67 and 1.25 V vs SCE, respectively.^12,18^ The presence of substrates changes the cyclic voltammetry at the Fe­(IV/V) redox couple in a manner characteristic of mass transport-limited electrocatalysis.^12,18^ In contrast, although well-defined Fe­(II/III) redox couples are observed for pentadentate pyridylamine ligand-based complexes (e.g., TPA, N4Py, and bispidine), redox waves assignable to the Fe­(III/IV) couples are not observed. Their absence has been ascribed to slow heterogeneous electron transfer kinetics between the LFe­(III)–OH species and the electrode.^19,20^ Alternative approaches to determining these redox potentials have been applied over the past decade, in particular, redox titrations and bulk electrolysis/spectroelectrochemistry, with redox potentials ranging from 0.4 to 1.3 V vs SCE (Scheme 1 and Table S1).^19–25^

**Scheme 1:**
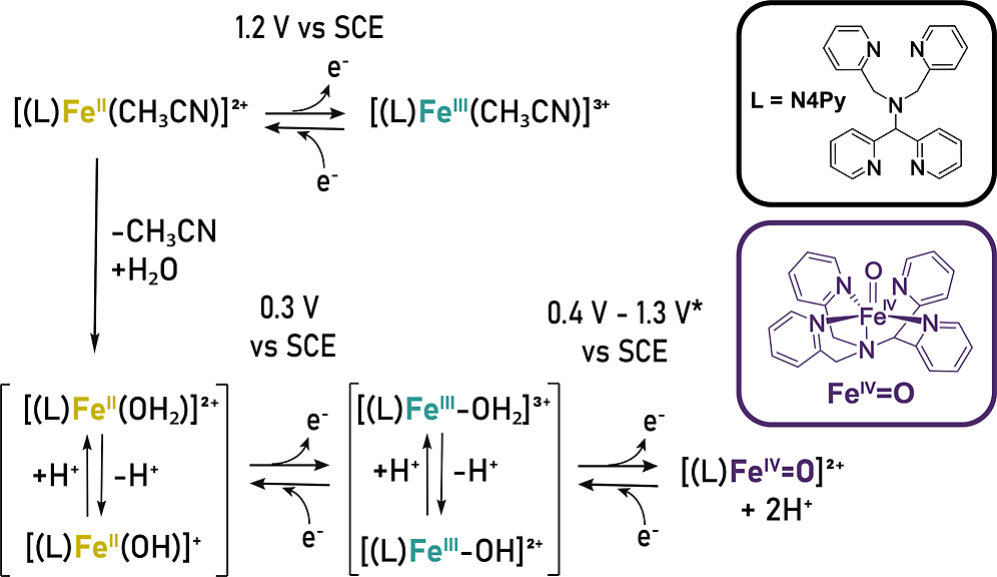
Redox Processes of ${\left[\left(N_{4}Py\right)Fe\left(II\right)\left({CH}_{3}CN\right)\right]}^{2+}$ , where L = N_4_Py, with the Main Ligand Exchange Reactions and Reported Potentials for the Fe­(III)/Fe­(IV) Redox Couple^a^

a *References 19–25 .

Here, we show that high-speed surface-enhanced Raman scattering (SERS) spectroscopy coupled with cyclic voltammetry can reveal Fe­(III/IV) redox potentials of the N5-bound complex ${\left[\left(N_{4}Py\right)Fe\left(II\right)\left({CH}_{3}CN\right)\right]}^{2+}$ (where N_4_Py is 1,1-bis­(pyridin-2-yl)-*N*,*N*-bis­(pyridin-2-ylmethyl)­methanamine),^26–29^ which are not manifested by redox waves in the cyclic voltammetry of the complex. The surface sensitivity achieved with SERS matches well with the electrode solution interface where the concentrations of species in various redox states are determined by overpotential (i.e., through the Nernst equation, Figure 1). Furthermore, the spectroscopic data indicate specific interaction between the Fe­(IV)=O species formed and the electrode surface. Comproportionation will deplete the diffusion layer of the Fe­(II) species ${\left[\left(N_{4}Py\right)Fe\left(II\right)\left({OH}_{2}\right)\right]}^{2+}$ , and other reactions, such as formation of dinuclear Fe­(III) complexes, will reduce the current obtained in the oxidation of Fe­(III) to Fe­(IV) species; however, the absence of a clear redox wave is most likely due to a low rate of heterogeneous electron transfer possibly due to the limited number of proton accepting sites on the electrode surface.

**Figure 1:**
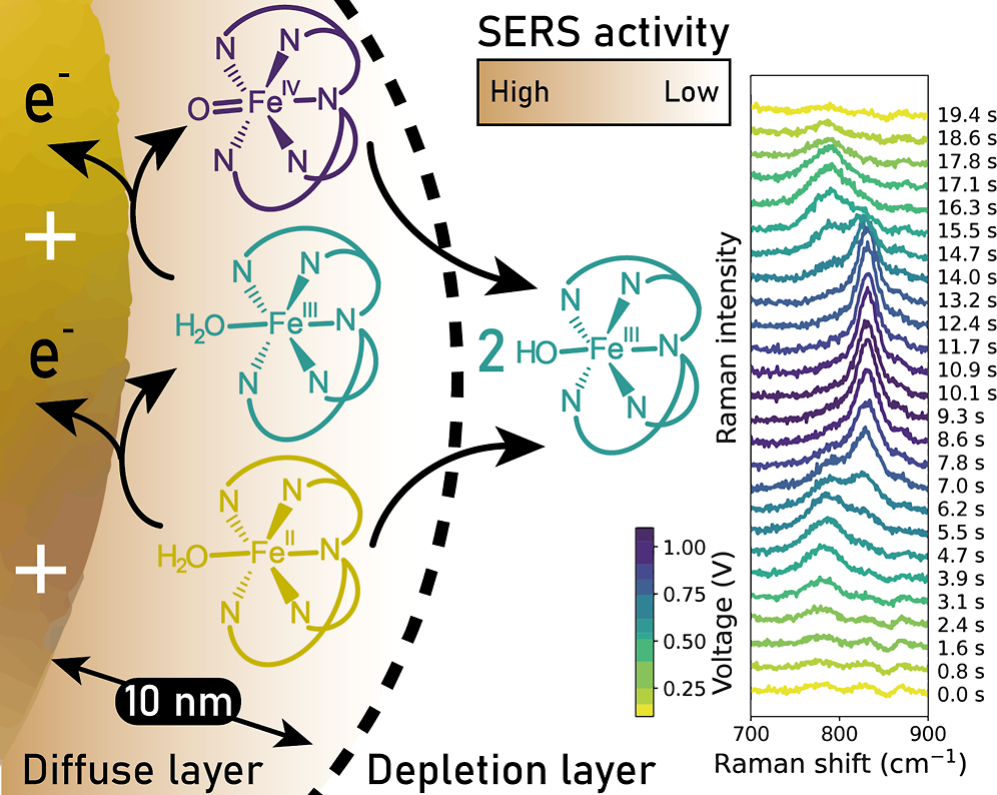
Electrochemical oxidation of ${\left[\left(N_{4}Py\right)Fe\left(II\right)\left({OH}_{2}\right)\right]}^{2+}$ from the Fe­(II) to Fe­(III) and then the Fe­(IV)=O state occurs at the electrode with the same distance dependence as surface enhancement of Raman scattering (right). Fe­(IV)=O diffusing away from the electrode comproportionates with Fe­(II)­OH_2_ in the depletion layer.

${\left[\left(N_{4}Py\right)Fe\left(II\right)\left({CH}_{3}CN\right)\right]}^{2+}$ was developed as a functional model for the antibiotic Fe-bleomycin and is one of the more widely studied of the N5-bound complexes.^26–29^ [(N_4_Py)­Fe­(IV)=O]^2+^ was one of the first examples of a crystallographically characterized nonheme Fe­(IV)=O species, and since then, many structural analogues have been reported by Que, Nordlander, and co-workers.^5,30^ These Fe­(IV)=O species are able to oxidize organic substrates^4^ and can be generated using chemical oxidants, such as *m*-CPBA,^31^ iodosylbenzene,^5^ and NaOCl,^32^ as well as electrochemically through bulk electrolysis.^19,20,22^ Despite this, determining the Fe­(III/IV) redox potential has proven challenging due to a strong dependence on the solvent and equilibria. UV/vis absorption spectropotentiometric titration,^20^ cyclic voltammetry,^21^ and UV/visible absorption spectropotentiometry^22^ have been applied to establish a Fe­(III)–OH to Fe­(IV)=O redox potential (Scheme 1). Reported values (vs SCE) are +1.24 V^22^ and +1.3 V^20^ in CH_3_CN with water, *E*_1/2_ = +0.41 V in water.^21^

Spectroelectrochemical techniques, such as UV/vis absorption spectroelectrochemistry, are useful in the study of these complexes due to the distinct NIR absorption band (λ_max_ = 690 nm in CH_3_CN with water) of the Fe­(IV)=O species. However, heterogeneous electron transfer^33^ requires that the redox active molecules approach to within a nanometer of the electrode double layer, and it is in this region that the concentration of the redox product is determined by overpotential. Concentrations in the depletion layer are impacted by mass transport-limited reactions, in particular, comproportionation, and it is this region (the depletion or Nernst diffusion layer) that is probed by most spectroscopies in spectroelectrochemical experiments. Since equilibration between the electrode interface and solution is mass transport-limited, typically by diffusion only, the response to changes in electrode potential is delayed and impacted by other reactions, not least comproportionation. In such cases, the species formed at the electrode never reach the bulk and are therefore not observed.

Confocal microspectroscopy enables study of changes at the electrode solution interface; however, the achievable confocal depth is still orders of magnitude greater (>2 μm) than the diffuse layer (<10 nm).^34–36^ When combined with SERS,^37–40^ however, selective observation of species at the diffuse layer is possible since the distance dependence for surface enhancement (<1–2 nm)^41,42^ corresponds well with the region in which electron transfer between the electrode and species in solution takes place.^33^ Indeed, the combination of electrochemistry and SERS (EC-SERS), or tip-enhanced Raman spectroscopy,^43,44^ has been used extensively in fields ranging from redox proteins adsorbed to electrodes,^45,46^ biological^47^ and quantitative analysis,^48–51^ to the study of electrochemical processes.^52–56^ Gold provides both the rough surface (i.e., hot spots^37^) needed for surface enhancement and acts as an electrode, which, in the present study, allows for transient species formed at the electrode to be studied selectively on time scales relevant to cyclic voltammetry (tens of milliseconds).^36^

## Results and Discussion

The cyclic voltammetry of ${\left[\left(N_{4}Py\right)Fe\left(II\right)\left({CH}_{3}CN\right)\right]}^{2+}$ in CH_3_CN and H_2_O was reported earlier.^57^ The Fe­(II)/Fe­(III) redox process is quasi-reversible depending on the solvent and pH due to exchange of the 6th ligand (e.g., CH_3_CN, Cl^–^, H_2_O, etc., Scheme 1). However, the Fe­(II)­(CH_3_CN) → Fe­(III)­(CH_3_CN) of ${\left[\left(N_{4}Py\right)Fe\left(II\right)\left({CH}_{3}CN\right)\right]}^{2+}$ redox couple is at 1.2 V,^57^ which is higher than the expected Fe­(III)­(OH_2_/OH) → Fe­(IV)=O redox potential.

${\left[\left(N_{4}Py\right)Fe\left(II\right)\left({CH}_{3}CN\right)\right]}^{2+}$ undergoes immediate reversible solvolysis in water to form ${\left[\left(N_{4}Py\right)Fe\left(II\right)\left(OH\right)\right]}^{+}$ and ${\left[\left(N_{4}Py\right)Fe\left(II\right)\left({OH}_{2}\right)\right]}^{2+}$ (p*K*_a_ = 4.5),^57^ which are oxidized at less positive potentials than ${\left[\left(N_{4}Py\right)Fe\left(II\right)\left({CH}_{3}CN\right)\right]}^{2+}$ . The *E*_1/2_ of the Fe­(II/III) redox couple is dependent on pH, ranging from −0.2 V (pH > 10) to 0.4 V (pH < 2) vs SCE.^57^ The less positive redox potential of the Fe­(II)­(OH_2_/OH) → Fe­(III)­(OH) couple enables quantitative conversion to ${\left[\left(N_{4}Py\right)Fe\left(III\right)\left(OH\right)\right]}^{2+}$ by bulk electrolysis at 0.56 V vs SCE (at pH 6.5). Hence, in water, Fe­(III)­(OH_2_/OH) → Fe­(IV)=O can be addressed subsequently. The cyclic voltammetry of ${\left[\left(N_{4}Py\right)Fe\left(II\right)\left({CH}_{3}CN\right)\right]}^{2+}$ in aqueous KNO_3_ at a gold, an electrochemically roughened gold (Figure 2), a GC, and a Pt electrode (Figure S1) all show the expected Fe­(II)/Fe­(III) redox couple (*E*_1/2_ = 0.27 V vs SCE).^57^ At the glassy carbon and platinum electrodes, further redox processes are not observed until the onset of solvent oxidation. With the gold electrode (Figure 2), the electrochemically irreversible oxidation of gold (Au/AuO), with an onset potential of 0.9 V vs SCE (depending on pH, see Figure S2), and subsequent reduction at 0.65 V vs SCE on the return cycle is observed also. The *I*_p,c_ of the AuO reduction wave increased with an increase in switching potential, as expected. Roughening of the gold electrode (in preparation for EC-SERS measurements) results in an earlier onset of Au/AuO oxidation due to the higher surface energy (Figure 2). A redox wave assignable to a Fe­(III)/Fe­(IV) redox couple is not observed at any of the electrodes.

**Figure 2:**
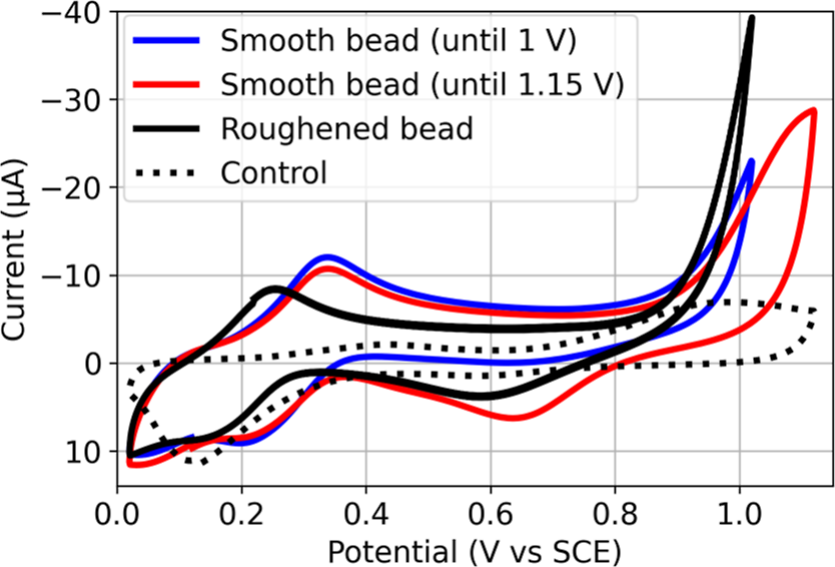
Cyclic voltammogram of ${\left[\left(N_{4}Py\right)Fe\left(II\right)\left({CH}_{3}CN\right)\right]}^{2+}$ (0.5 mM) in H_2_O at pH 4–5 at a smooth Au electrode (blue), a smooth Au electrode to a more positive potential (red), an electrochemically roughened Au electrode (black), and a control at a roughened Au electrode in the absence of ${\left[\left(N_{4}Py\right)Fe\left(II\right)\left({CH}_{3}CN\right)\right]}^{2+}$ (black, dotted). In 0.1 M KNO_3_ (aq) at pH 4–5 (adjusted with HNO_3_). Scan rate 0.1 V s^–1^, RE: Ag/AgCl, and CE: Pt.

### SERS of ${\left[\left(N_{4}Py\right)Fe\left(II\right)\left({CH}_{3}CN\right)\right]}^{2+}$ at a Roughened Gold Electrode

The Raman spectrum of a 2 mM solution of ${\left[\left(N_{4}Py\right)Fe\left(II\right)\left({CH}_{3}CN\right)\right]}^{2+}$ in KNO_3_ (aq) shows only the Raman band of the nitrate anion at 1049 cm^–1^ (Figure 3 top, black). Surface enhancement is readily apparent when the Raman microscope is focused on a roughened gold electrode immersed in the solution (Figure 3 top, red). The SERS spectrum of ${\left[\left(N_{4}Py\right)Fe\left(II\right)\left({OH}_{2}\right)\right]}^{2+}$ is, as expected, similar to the nonresonant Raman spectrum of solid ${\left[\left(N_{4}Py\right)Fe\left(II\right)\left({OH}_{2}\right)\right]}^{2+}$ with small shifts in individual bands (Figure 3 bottom, black).^58^ Raman bands at 1595, 1570, 1159, and 1020 cm^–1^ are assigned to pyridyl-based modes based on the resonance Raman spectra at 473 nm^57^ and are similar in the SERS and resonance Raman spectrum (Figure 3 bottom, blue). Bands at 1465, 1375, and 1220 cm^–1^ correspond to the C–H vibrational modes of the alkyl amine backbone, and the mode at 1205 cm^–1^ corresponds to the alkyl amine backbone. Bands at 870, 828, 762, 686, 652, and 633 cm^–1^ are similar between the SERS and the Raman spectra also.^59^

**Figure 3:**
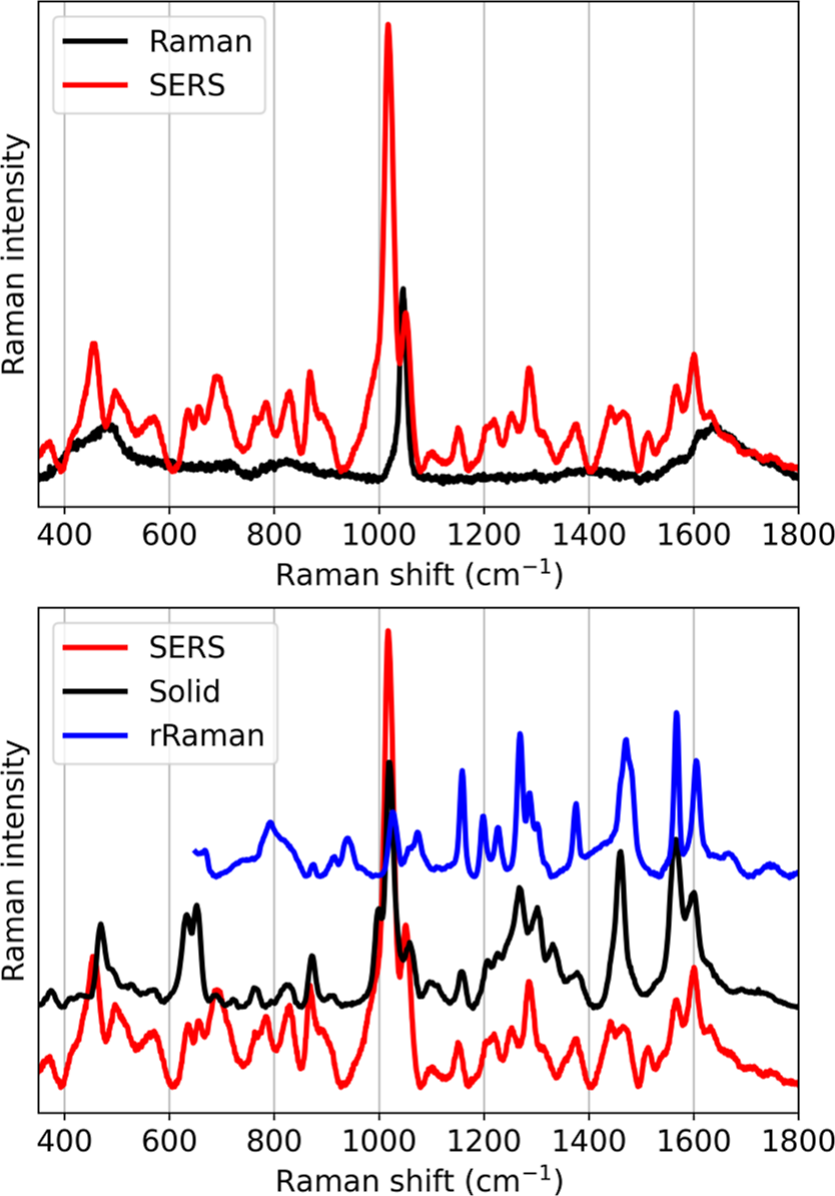
Raman spectra (top) SERS (red) and nonresonant Raman (black) spectrum of ${\left[\left(N_{4}Py\right)Fe\left(II\right)\left({CH}_{3}CN\right)\right]}^{2+}$ (2 mM) in 0.1 M KNO_3_ (aq) in solution at λ_exc_ 785 nm, (bottom) SERS (λ_exc_ 785 nm, red), resonance Raman spectrum (λ_exc_ 473 nm at pH 5, blue) in H_2_O, and solid-state Raman spectrum (λ_exc_ 785 nm, black) of the sample prepared by drop-cast deposition.

The position of Raman bands of ${\left[\left(N_{4}Py\right)Fe\left(II\right)\left({OH}_{2}\right)\right]}^{2+}$ is highly sensitive to conditions, as apparent in both differences in the SERS spectra recorded at pH 4.5 and pH 6.5 (Figure S3); however, these differences are similar to variations seen between measurements under the same conditions with different gold beads (Figure S4). It should be noted that during cyclic voltammetry, the pH at the electrode can vary considerably even in buffered aqueous solutions,^56^ and indeed, nonresonant Raman spectra of solid ${\left[\left(N_{4}Py\right)Fe\left(II\right)\left({OH}_{2}\right)\right]}^{2+}$ prepared by drop-cast deposition Raman methods^60^ show sample to sample variations (Figure S6).

### Concurrent Cyclic Voltammetry and SERS Spectroscopy

SERS spectra of ${\left[\left(N_{4}Py\right)Fe\left(II\right)\left({OH}_{2}\right)\right]}^{2+}$ were recorded concomitant with cyclic voltammetry over two cycles between 0.1 and 1.1 V vs SCE (Figures 4 and 5). Polarization of the electrode to positive potentials resulted in reversible changes to the SERS spectrum with the intensity of bands between 1000 and 1700 cm^–1^ decreasing and bands between ∼700 and 900 cm^–1^ appearing. Three distinguishable SERS spectra were observed at ∼0.1, 0.5, and 1.0 V vs SCE. During subsequent cycles, the potential sweep was paused at these potentials for a longer period to increase the signal-to-noise ratios of the spectra (Figure 5A).

**Figure 4:**
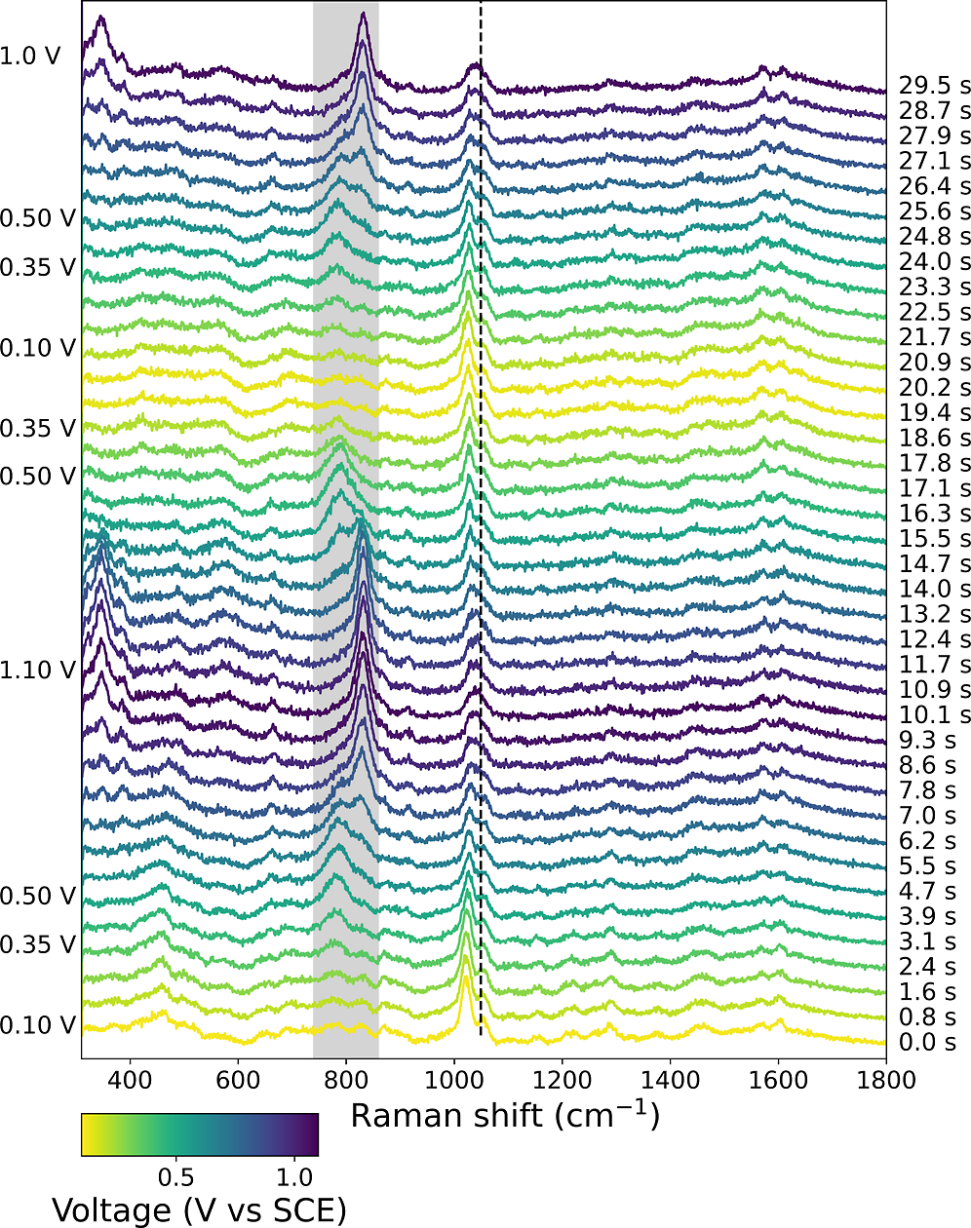
SERS spectra of ${\left[\left(N_{4}Py\right)Fe\left(II\right)\left({CH}_{3}CN\right)\right]}^{2+}$ (2 mM) recorded during cyclic voltammetry. The potential and time are indicated by color (0.1 V to 1.1 V vs SCE) and indicated on the left and right, respectively. 0.1 M KNO_3_ (aq), WE: gold bead, RE: Ag/AgCl, CE: Pt, and scan rate: 0.1 V s^–1^, SERS spectra recorded with 0.5 s acquisitions at λ_exc_ 785 nm. Spectra were normalized to the NO_3_^–^ band at 1049 cm^–1^, indicated with a dotted black line.

**Figure 5:**
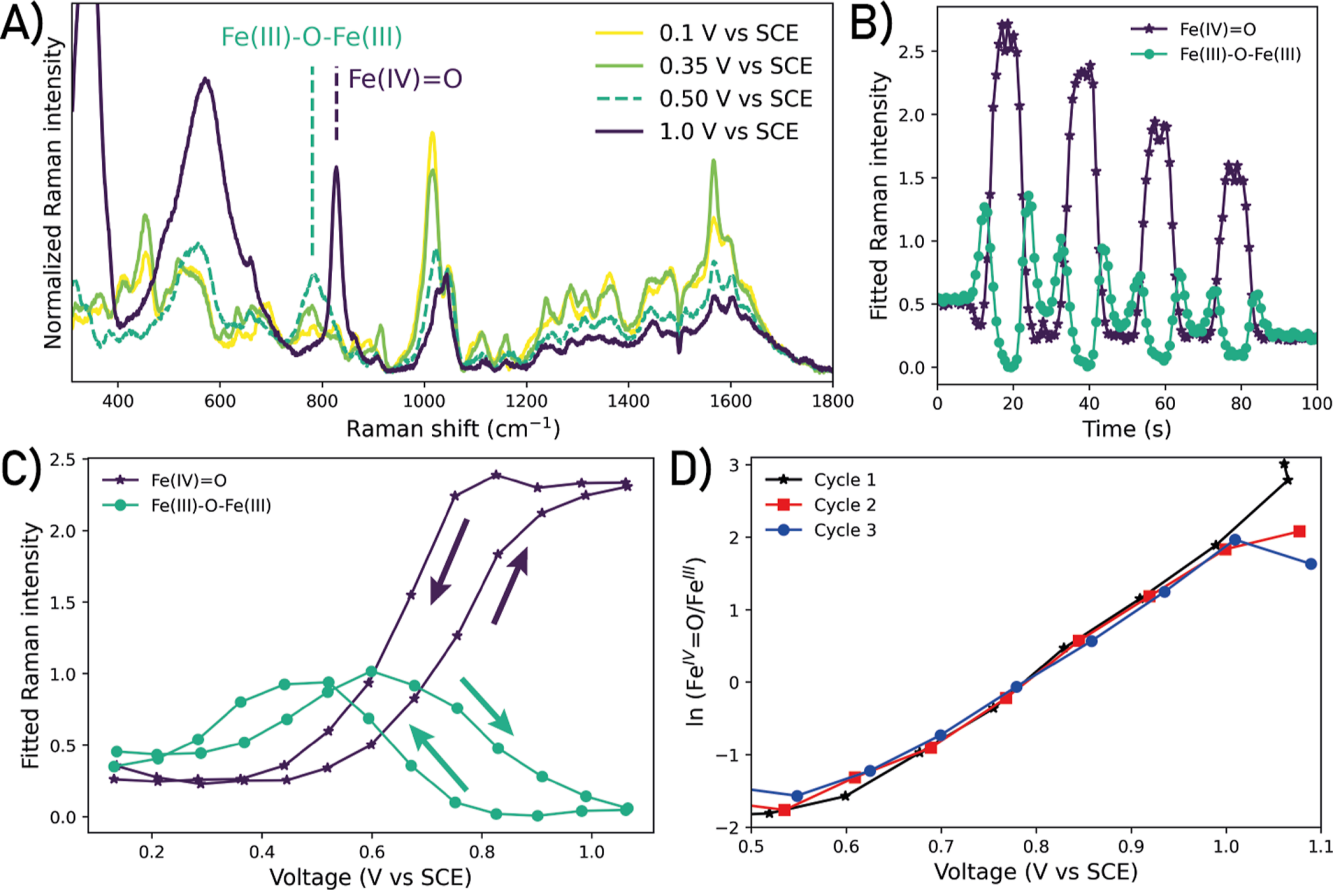
(A) SERS spectra of ${\left[\left(N_{4}Py\right)Fe\left(II\right)\left({OH}_{2}\right)\right]}^{2+}$ in H_2_O recorded with the electrode held at the potentials indicated; fitted surface-enhanced Raman intensity from Fe­(III)­O–Fe­(III) and Fe­(IV)=O vs (B) time and (C) voltage, respectively. (D) Fitted SERS intensities of three forward scans from Fe­(III)–O and Fe­(IV)=O plotted as ln­(Fe^IV^ = O/Fe^III^) vs potential. See the Supporting Information for further discussion of fitting. Conditions: 2 mM [(N_4_Py)­Fe­(II)­(CH_3_CN)]­(OTf)_2_, 0.1 M KNO_3_ (aq). SERS spectra recorded by accumulation of five 5 s acquisitions at 785 nm, scan rate 0.1 V s^–1^, WE: gold bead, RE: Ag/AgCl, and CE: Pt. Spectra were normalized on the NO_3_^–^ band at 1049 cm^–1^.

As the potential is increased past 0.35 V vs SCE, a band at 780 cm^–1^ and past 0.70 V vs SCE at 828 cm^–1^ appear. Beyond 0.90 V vs SCE, most of the intensity, except for that of the Au–O band, is lost. The bands are recovered again as the potential is swept negatively. A strong broad band appears at 570 cm^–1^ as the potential increases past 0.9 V vs SCE for a longer time (Figure 5A), which is assigned to a AuO stretch,^53,61–63^ as it is present without ${\left[\left(N_{4}Py\right)Fe\left(II\right)\left({CH}_{3}CN\right)\right]}^{2+}$ , and shows an isotope shift with ^18^O labeled water (vide infra).

The individual bands in the SERS spectra (Figure 4) between 730 and 870 cm^–1^ were resolved using multivariate curve resolution to isolate the contributions from the bands at 780 cm^–1^ and 828 cm^–1^ assigned to the Fe­(III)–O–Fe­(III) and the Fe­(IV)=O species, respectively (see Figure S7 and Supporting Information for details). The band at 780 cm^–1^ is consistent with reported heme and nonheme Fe­(III)–O–Fe­(III) dinuclear complexes,^64,65^ whereas the Fe­(III)–OH complexes are expected to have an Fe­(III)–O stretching mode closer to 560 cm^–1^ (e.g., for Fe­(III)–OMe, the corresponding band is at 554 cm^–1^ ^66^). These fitted SERS bands and the contributions of each to the spectrum (from Figure 4) were plotted against time (Figure 5B) and potentials (Figure 5C) at which they were recorded. The band at 780 cm^–1^, assigned to [(N_4_Py)­Fe­(III)–O–Fe­(III)­(N_4_Py)]^4+^, appears at 0.4 V vs SCE and increases until ca. 0.6 V vs SCE, where after it decreases again. Concomitant with the latter decrease, a band at 828 cm^–1^ appears and reaches a maximum at ca. 0.9 V vs SCE. This band was assigned to the Fe­(IV)=O species, and it remains constant until the potential sweep is reversed at 1.1 V vs SCE. The band intensity decreases again at ca. 0.8 V vs SCE. The band assigned to a ν_asymm_Fe­(III)–O–Fe­(III) mode (780 cm^–1^) appears only at a slightly more positive potential (ca. 0.4 V vs SCE) than the *E*_1/2_ of the Fe­(II)/Fe­(III) redox wave in the corresponding cyclic voltammogram (*E*_1/2_ = 0.3 V vs SCE, Figure S9) due to lag time between the current response in the cyclic voltammogram and the moment the band intensity rises above the noise level. Furthermore, the ratio in SERS intensities of the bands assigned to Fe­(IV)=O and Fe­(III)–O–Fe­(III) stretching modes show a log linear $\left(\ln\left(\frac{Fe\left(IV\right)}{Fe\left(III\right)}\right)vs\;E\right)$ dependence on potential as expected from the Nernst eq (Figure 5D). Based on the potential at which the bands assigned to Fe­(IV)=O and Fe­(III)–O–Fe­(III) are each at half-maximum intensity, we estimated the Fe­(III)/Fe­(IV) redox potential to be 0.7–0.85 V vs SCE (see the Supporting Information for discussion).

It is of note that the 780 cm^–1^ band is less intense in the SERS spectrum recorded when the potential was held at 0.5 V vs SCE (Figure 5A) compared to that observed during cyclic voltammetry (Figure S11). The differences in intensity and band shape may be due to differences in pH at the electrode under the different conditions and/or formation of Fe­(III)–O–Fe­(III) dimers, which advocate the importance of the time-resolved approach taken in this work.

On the return cycle from 0.8 V vs SCE to negative potentials, the band at 780 cm^–1^ reappears at ca. 0.7 V vs SCE, increases until ca. 0.4 V vs SCE, and then decreases again. The intensities of the bands at 828 cm^–1^ and 780 cm^–1^ decrease slightly with each cycle (Figure 5B), likely due to the loss of the roughening of the gold bead during potential cycling with concomitant loss of SERS efficiency as a result. Concurrent SERS/cyclic voltammetry of ${\left[\left(N_{4}Py\right)Fe\left(II\right)\left({CH}_{3}CN\right)\right]}^{2+}$ in methanol, over two cycles between 0.1 and 1.1 V vs SCE (Figures S12–S14), shows similar behavior as in H_2_O, although the spectra of ${\left[\left(N_{4}Py\right)Fe\left(III\right)\left({OCH}_{3}\right)\right]}^{2+}$ and ${\left[\left(N_{4}Py\right)Fe\left(II\right)\left({HOCH}_{3}\right)\right]}^{2+}$ are almost indistinguishable with the exception of the band at 554 cm^–1^ assigned to the Fe­(III)–OCH_3_ stretch of ${\left[\left(N_{4}Py\right)Fe\left(III\right)\left({OCH}_{3}\right)\right]}^{2+}$ .^66^

### Comparison of Solid-State Raman and SERS Spectra of [(N_4_Py)­Fe­(IV)=O]^2+^

Although shifted by 13 cm^–1^, with respect to the ν_(str,Fe=O)_ band of Fe­(IV)=O observed in the solid state and solution at 841 cm^–1^,^19,32^ the SERS spectrum recorded at 0.9 V vs SCE (Figure 6 black and red, respectively) shows a band that is consistent with a ν_(str,Fe=O)_ band at 828 cm^–1^ (vide infra).

**Figure 6:**
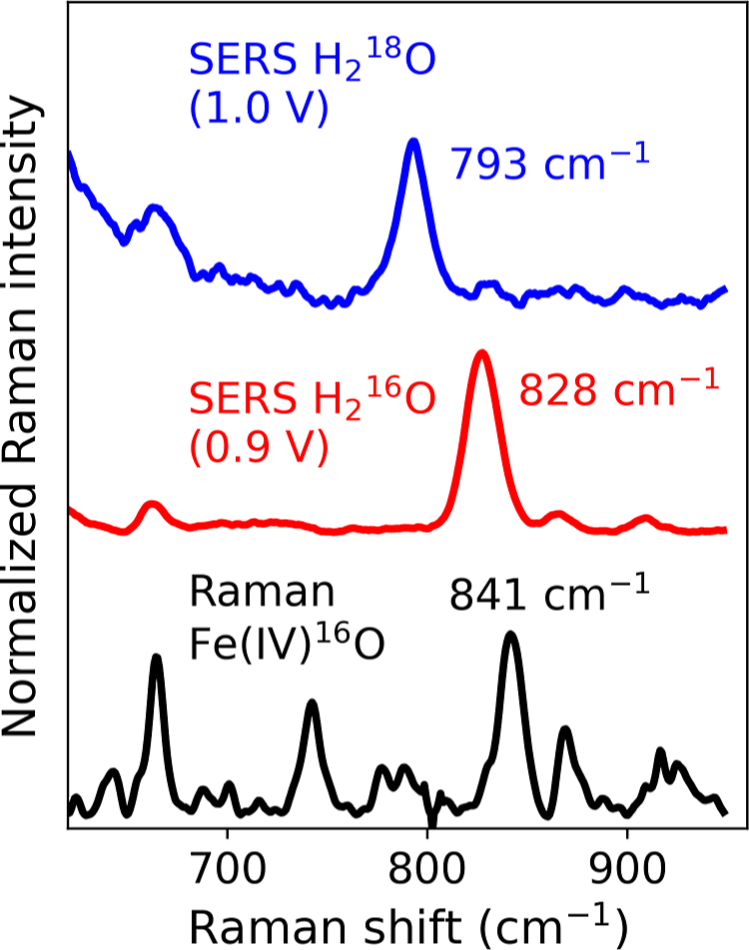
Raman spectrum of [(N_4_Py)­Fe­(IV)=O]^2+^ (black) compared to a SERS spectrum of ${\left[\left(N_{4}Py\right)Fe\left(II\right)\left({OH}_{2}\right)\right]}^{2+}$ at 0.9 V vs SCE in H_2_O (red) and compared to a SERS spectrum of ${\left[\left(N_{4}Py\right)Fe\left(II\right)\left({OH}_{2}\right)\right]}^{2+}$ at 1.0 V vs SCE in H_2_^18^O (blue). SERS and Raman spectra were recorded at λ_exc_ 785 nm, with, respectively, 5 s acquisition and 5 accumulation and 50 s acquisition and 20 accumulations.

### H_2_^18^O Labeling

SERS spectra of ${\left[\left(N_{4}Py\right)Fe\left(II\right)\left({OH}_{2}\right)\right]}^{2+}$ at a roughened gold bead in H_2_^18^O/KNO_3_ showed similar potential-dependent changes as observed in H_2_^16^O/KNO_3_ (Figure 6, red and blue, respectively, and Figure S15). The band at 828 cm^–1^ observed at 0.9 V vs SCE, assigned to the Fe­(IV)=O stretch in H_2_^16^O, is shifted by 35 cm^–1^ to 793 cm^–1^ in H_2_^18^O, which is the expected isotope shift for Fe­(IV) = ^16^O/^18^O. The band at 780 cm^–1^ in the spectrum in H_2_^16^O recorded at 0.35 V vs SCE and at 763 cm^–1^ in H_2_^18^O at 0.3 V vs SCE (Figure S15) is assigned to an Fe­(III)–O stretch of either an Fe­(III)–O–Fe­(III) dinuclear complex or a mononuclear Fe­(III)–OH complex,^25,67^ as this band is present only at potentials where the Fe­(III) species should be the main species present at the electrode. The band assigned to a Au–O stretch,^61,62^ which appears at positive potentials, is shifted by 30 cm^–1^ from 570 cm^–1^ to 540 cm^–1^ (Figure S16). From these data, the bands observed at 0.35 V versus SCE and at 0.9 V versus SCE can be assigned to Fe­(III)–O–Fe­(III) and Fe­(IV)=O species, respectively. The Fe­(IV)=O stretching band is relatively strong in the SERS spectrum compared with other bands (e.g., ligand modes), which arises from additional weak resonance enhancement (λ_max_ for Fe­(IV)=O is 695 nm) at λ_exc_ 785 nm, i.e., surface-enhanced resonance Raman scattering.^36^

### DFT Calculations

The interaction between the surface plasmon of the roughened gold surface and the complex enhances the latter’s Raman scattering at concentrations where one would normally not be able to observe the Raman scattering from the complex. The interaction of the complex with the surface, however, can alter its environment compared to that in the bulk of the solvent and even its structure if specific adsorption/chemisorption occurs. Such interactions can rationalize the experimentally observed shift in the Raman band. Density functional theory (DFT) methods employing gold clusters/nanoparticles (55 atoms), shown earlier to represent reasonably well a gold nanoparticle/adsorbate interaction effect on Raman shift,^51^ were used to model the impact of interactions between the gold nanoparticle and the Fe­(IV)=O species formed electrochemically. It is difficult to estimate the effect of binding of the molecule to the roughened gold surface solely from the change in Raman shift due to polarization of the SERS active electrode, and hence, here a qualitative comparison is made only. The Raman shift computed for the Fe­(IV)=O band (846 cm^–1^) shifts to 726 cm^–1^ when bound to a 55 atom nanoparticle, i.e., in the same direction as observed experimentally (Figure 7, see the Supporting Information for details). The computed shift is almost certainly an overestimation, as binding to the gold nanoparticle results in 0.18 electron transferring from the complex to the nanoparticle and a 0.05 Å increase in bond length. This increase is consistent with the weakening of the Fe­(IV)=O bond. Such binding via the oxo ligand is observed also between ${\left[\left(N_{4}Py\right)Fe\left(III\right)\left(OH\right)\right]}^{2+}$ and Ce^IV^ by Que et al., forming a reversible Fe­(III)–O–Ce­(IV) complex.^25^

**Figure 7:**
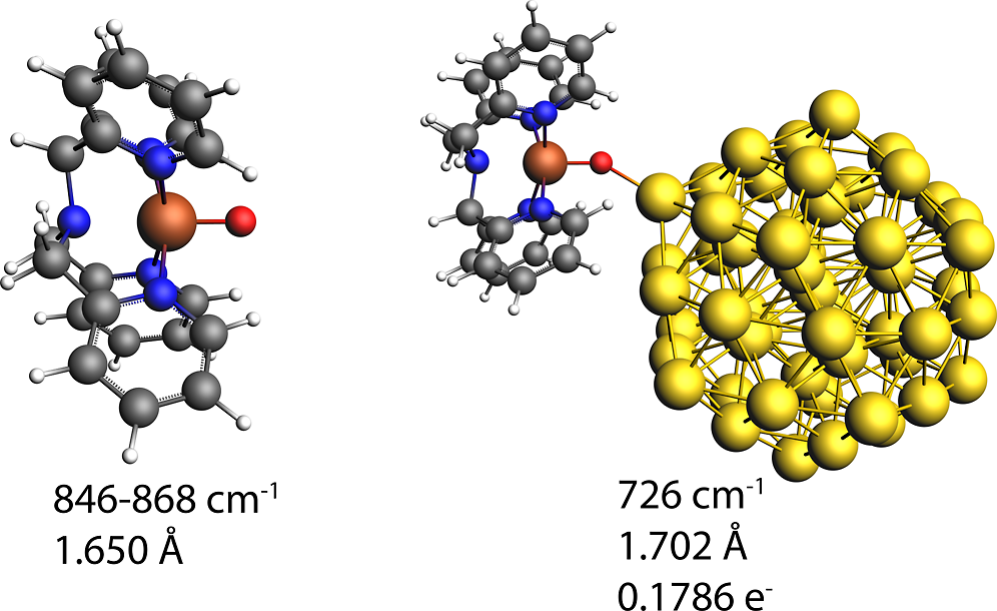
Optimized geometries, Fe–O vibrational mode shifts, Fe–O bond lengths, and electron density transferred for the free [(N_4_Py)­Fe­(IV)=O]^2+^ complex (PBE/TZ2P) and the complex adsorbed on a gold nanoparticle (PBE/TZ2P, 55 atoms, icosahedral geometry, see the Experimental Details section).

## Conclusions

In the present study, high-speed Raman spectroscopy coupled with surface enhancement provides for direct observation of the species present at the electrode surface and allows for determination of the potential of the Fe­(III)/Fe­(IV) redox couple of ${\left[\left(N_{4}Py\right)Fe\left(II\right)\left({OH}_{2}\right)\right]}^{2+}$ . The potential, 0.70–0.85 V vs SCE, is in agreement with that determined by titration with chemical oxidants by Que and co-workers earlier,^25^ and other methods used to determine the redox potential of the iron complex are erroneous due to the solvent-dependent ligand exchange and comproportionation reactions that are central to the (electro)­chemistry of ${\left[\left(N_{4}Py\right)Fe\left(II\right)\left(X\right)\right]}^{n+}$ . The present data also shed light on possible reasons why a clear Fe­(III)/Fe­(IV) redox wave is not observed in the cyclic voltammetry of ${\left[\left(N_{4}Py\right)Fe\left(II\right)\left({OH}_{2}\right)\right]}^{2+}$ in contrast to, for example, the Fe–TAML complexes. The shift in the Fe­(IV)=O stretching band in the SERS spectrum suggests, based on DFT calculations, chemisorption or at least specific adsorption to the electrode. This factor combined with comproportionation reactions and possible formation of dinuclear Fe­(III)–O–Fe­(III) species likely compounds the impact of a low rate of heterogeneous electron transfer between the complex and the electrode and hence suppresses the redox wave in the cyclic voltammetry. The approach taken here shows that high-speed Raman spectroscopy coupled with electrochemistry can provide for direct readout of surface concentrations of redox active species. Furthermore, it overcomes limitations that slow electron transfer kinetics present in observing redox processes and provides essential thermodynamic data in the study of catalysts that show PCET/HAT reactivity.

## Experimental Details

All solvents and reagents were obtained from commercial suppliers and used as received. [(N_4_Py)­Fe­(II)­(CH_3_CN)]­(PF_6_)_2_ was available from earlier studies.^32^

### Physical Methods

SERS studies were carried out at 785 nm using a 20× long working distance objective on a BX-51 microscope directed horizontally from the objective turret using a gold mirror onto a microcuvette (Hellma) containing the electrolyte and electrodes. Excitation was provided by an ONDAX LM-785 laser (75 mW at source) which was passed through a laser line clean up filter (CleanLine FS, Ondax), a $\frac{1}{2}\lambda$ retarder and polarizing beam splitter to control power followed by a second $\frac{1}{2}\lambda$ retarder to control polarization. The laser was combined with the optical path of the spectrometer with a dichroic mirror (45°) (Semrock Di02-R785) and directed to the microscope with gold mirrors. The Raman scattering passed through the dichroic mirror and a Rayleigh line rejection filter (Semrock BLP01-785R) and was focused with a 35 mm focal length plano convex lens into an Andor Kymera-193i spectrograph with a 600 lines/mm grating blazed at 750 nm and Andor idus-DU416A-LDC-DD CCD camera.

Raman spectra of drop-cast samples were recorded using a 50× long working distance objective on a BX-51 microscope. Excitation was provided by an ONDAX Mini-Benchtop Stabilized Lasers at 785 (500 mW at source) coupled to the microscope with a 100 μm multimode optical fiber. The excitation is passed through a laser line clean up filter and a dichroic mirror (45°) (Semrock Di02-R785) and directed to the microscope with dielectric mirrors. The Raman scattering passed through the dichroic mirror and a Rayleigh line rejection filter and was focused into a 100 μm multimode optical fiber connected to a Shamrock163i spectrograph with a 600 L/mm 830 nm blazed grating and a iVac-316-LD-DD CCD camera (Andor Technology). Spectra were acquired with an Andor Solis. Spectra were calibrated with polystyrene (ASTM E 1840).

Raman spectra were recorded at 473 (50 mW Cobolt Lasers) in solutions held in quartz cuvettes in a 180° back scattering arrangement. Dichroic mirrors were used to combine the excitation laser with the optical path of the spectrometer. A 2.5 cm diameter plano-convex lens (*f* = 7.5 cm) was used for both focusing the laser and collecting Raman scattering. The collimated Raman scattering was passed through the dichroic mirror and an appropriate long pass filter (Semrock) and focused by a 2.5 cm diameter plano-convex lens (*f* = 7.5 cm) into a Shamrock 300i spectrograph (Andor Technology) with a 1800 L/mm grating blazed at 500 nm or 600 L/mm at 800 nm and acquired with a Newton 970-BU EMCCD camera (Andor Technology) or a Zyla 4.2. The slit width was set to 50 or 100 μm. Data were recorded with spectral calibration performed using the Raman spectrum of cyclohexane following ASTM E 1840) and processed using Solis (Andor Technology) and Spectragryph.

Cyclic voltammograms were recorded by using a CH Instruments 670*e* potentiostat.

### Density Functional Theory

DFT calculations were performed using Amsterdam density functional in the Amsterdam Modeling Suite (version 2022.1).^68,69^ All calculations were done with the Perdew, Burke, Ernzerhof functional and TZ2P basis set,^70^ including solvation (water, COSMO solvation model).^71,72^ Molecular orbitals were expanded in an uncontracted set of Slater-type orbitals (STOs) of triple-ζ quality with double polarization functions (TZ2P).^73^ Core electrons were not treated explicitly during the geometry optimization (frozen core approximation). An auxiliary set of s, p, d, f, and g STOs was used to fit the molecular density and to represent the Coulomb and exchange potentials accurately for each SCF cycle. The PBE functional was chosen as it has been found in earlier studies^51,74–77^ to describe the properties of the gold nanoparticle well and describes the geometric features of the iron complex reasonably well also. While hybrid functionals would better describe the electronic states of the complex, the main focus of these calculations is the charge transfer between the complex and the nanoparticle and the resulting change in shifts in vibrational modes. The position of the lowest unoccupied molecular orbital of the nanoparticle has to be accurate in order to describe this charge transfer well, and hence, a functional such as PBE, which can accurately describe the metallic properties of the nanoparticle, is necessary.

Geometries of all possible spin states were optimized using adapted delocalized coordinates until the maximum gradient component was less than 10^–4^ au; the lowest energy states were selected for frequency analysis. Scalar relativistic corrections have been included self-consistently in all calculations by using the zeroth-order regular approximation. All calculations were done using the unrestricted Kohn–Sham scheme, and were done using the Becke grid of Good quality.

After the geometries were fully relaxed, frequency calculations were performed to confirm the ground-state structures and obtain frequencies of vibrational modes and infrared spectra. Mulliken population analysis was performed to evaluate atomic charges and electron transfer between the nanoparticles and the complex.^78^

The gold nanoparticle of 55 atoms (1.5 nm) was selected because in earlier studies,^79,80^ in which a range of sizes of nanoparticles were studied,^51^ it was determined that this size finds an optimum in size that allows it to be large enough to properly describe the charge transfer and being small enough to be computationally feasible. The nanoparticles in this range of sizes exist in both icosahedral and cuboctahedral forms, of which the icosahedral form used in this study was determined to be the most stable.

## Supplementary Material



## Notes

Additional spectroscopic and electrochemical data and details of MCR analysis (PDF)
