# Supplementary material for: Seeing an Unobservable Fe(III)/Fe(IV) Redox Process of the Nonheme Iron N4Py Complex by High-Speed Surface-Enhanced Raman Spectroelectrochemistry
Source: Inorg Chem. 2025 May 20;64(21):10549–57. doi: 10.1021/acs.inorgchem.5c01017 (PMC12135866; doi:10.1021/acs.inorgchem.5c01017)
Supplement: Supplementary file 1 [file ic5c01017_si_001.pdf]

Supporting information

Seeing an unobservable Fe(III)/Fe(IV) redox process of the non-heme iron N4Py complex by high-speed surface enhanced Raman spectroelectrochemistry

C. Maurits de Roo,[a] W. J. Niels Klement,[a] Daniel R. Duijnstee,[a] Aleksandar Staykov,[*,b] and Wesley R. Browne[*,a]

[a]Molecular Inorganic Chemistry, Stratingh Institute for Chemistry, Faculty of Science and Engineering, University of Groningen, Nijenborgh 3, 9747 AG, Groningen, The Netherlands

[b]International Institute for Carbon Neutral Energy Research (WPI-I_2CNER), Kyushu University, 744 Motooka, Nishi-ku, Fukuoka 819-0395, Japan

*alex@i2cner.kyushu-u.ac.jp, w.r.browne@rug.nl

Reported redox potentials of (N4Py)Fe(III)/(N4Py)Fe(IV)

Table S1: Reported redox potentials of $[(N_4Py)Fe(III)OH]^{2+}/[(N_4Py)Fe(IV)=O]^{2+}$

Potential (V) vs SCE	Solvent	Reference
1.3	MeCN	Que et al. ref 1
0.51	MeCN	Fukuzumi et al. ref 2
0.41	H_2O	Que et al. ref 3
0.94	MeCN	Que et al. ref 4
1.24	MeCN	Fukuzumi et al. ref 5
0.51	MeCN	Fukuzumi et al. ref 6
0.9-1.1	MeCN	Que et al. ref 7

Cyclic voltammetry at GC and Pt electrodes in H_2O

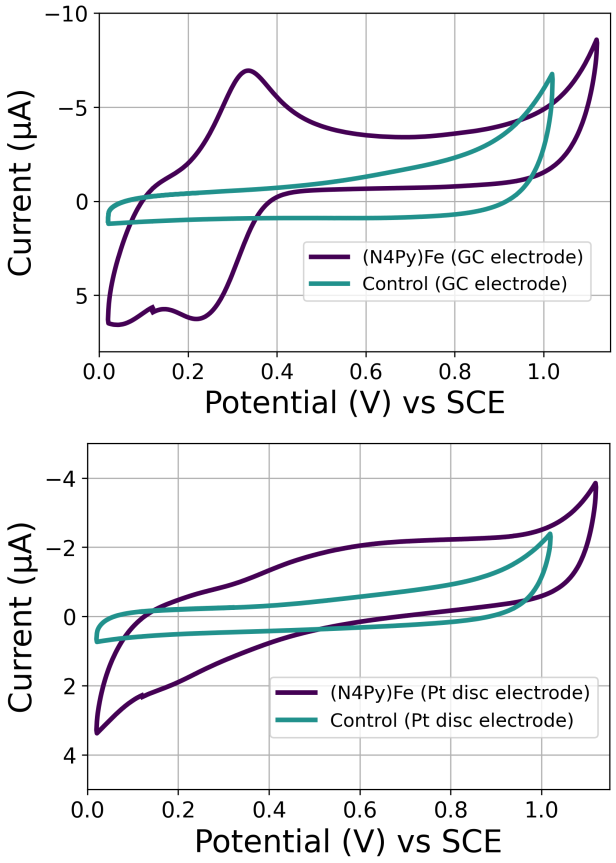

Figure S1: Cyclic voltammetry recorded at glassy carbon (top) and platinum disc (bottom) electrodes of $[(N_4Py)Fe(II)(CH_3CN)]^{2+}$ in H_2O. Conditions: 0.5 mM $[(N_4Py)Fe(II)(CH_3CN)](BF_4)_2$, 0.1 M KNO_3 in H_2O at pH 4-5 (HNO_3). 0.1 V s^{-1} scan rate, RE: Ag/AgCl, and CE: Pt.

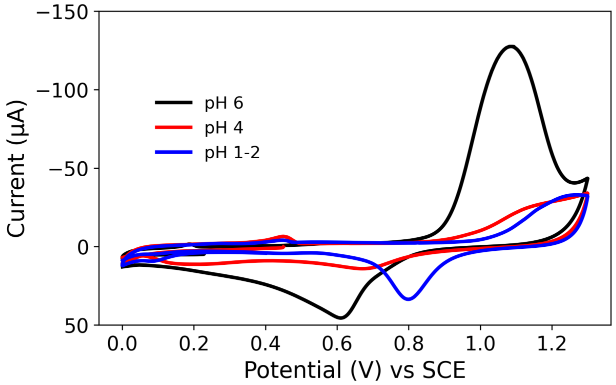

Figure S2: Cyclic voltammetry at a roughened gold bead in H_2O (0.1 M KNO_3) at different pH (adjusted by HNO_3): pH 6 (black), pH 4 (red), and pH 1-2 (blue). Roughened gold bead working electrode, SCE reference electrode, and Pt counter electrode.

Control experiments for SERS of $[(N_4Py)Fe(II)(CH_3CN)]^{2+}$ in H_2O

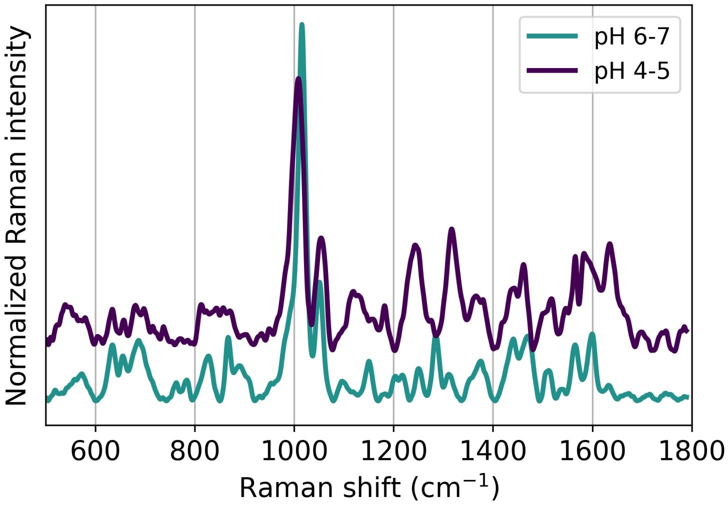

Figure S3: SERS of $[(N_4Py)Fe(II)(OH_2)]^{2+}$ at pH 6-7 compared to pH 4-5 (adjusted by HNO_3). Conditions: $[(N_4Py)Fe(II)(CH_3CN)](OTf)_2$ (2 mM), 0.1 M KNO_3 in H_2O. Spectra recorded by accumulation of five 5 s acquisitions at λ_{exc} 785 nm.

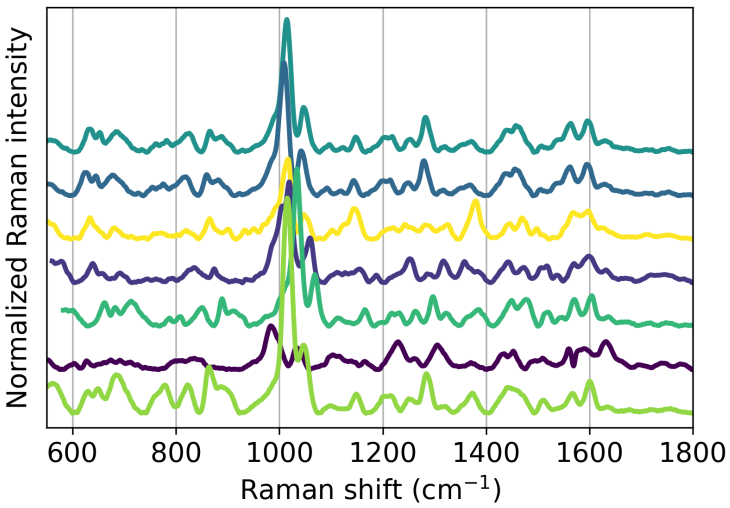

Figure S4: SERS spectra of $[(N_4Py)Fe(II)(OH_2)]^{2+}$ in H_2O under similar conditions at different roughened gold beads. Conditions: 2 mM $[(N_4Py)Fe(II)(CH_3CN)](OTf)_2$, 0.1 M KNO_3 in H_2O. Spectra recorded by accumulation of five 5 s acquisitions at λ_{exc} 785 nm.

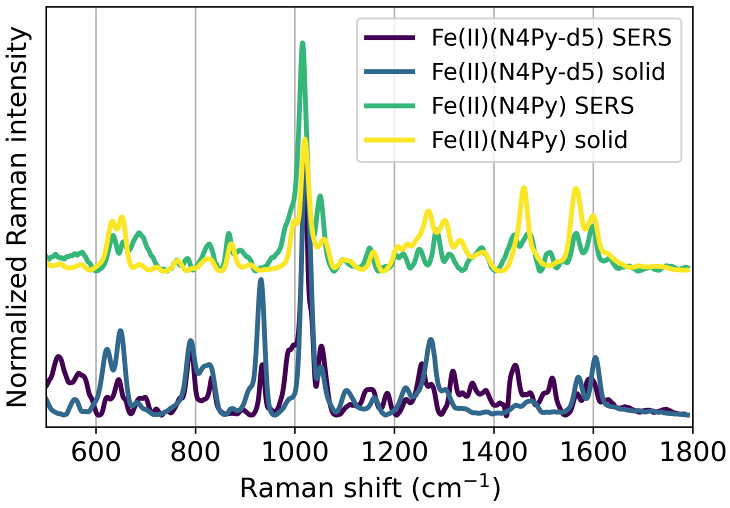

Figure S5: SERS and solid state Raman spectra (prepared by drop coat deposition) of $[(N_4Py{-}d_5)Fe(II)(OH_2)]^{2+}$ compared to $[(N_4Py)Fe(II)(OH_2)]^{2+}$. Conditions: $[(N_4Py{-}d_5)Fe(II)(CH_3CN)](PF_6)_2$ (1.7 mM), 0.1 M KNO_3 in H_2O. Spectra recorded by accumulation of five 5 s acquisitions at λ_{exc} 785 nm. Drop coat depostion analysis: $[(N_4Py{-}d_5)Fe(II)(CH_3CN)](PF_6)_2$ in H_2O at pH 7, drop cast on aluminum foil.

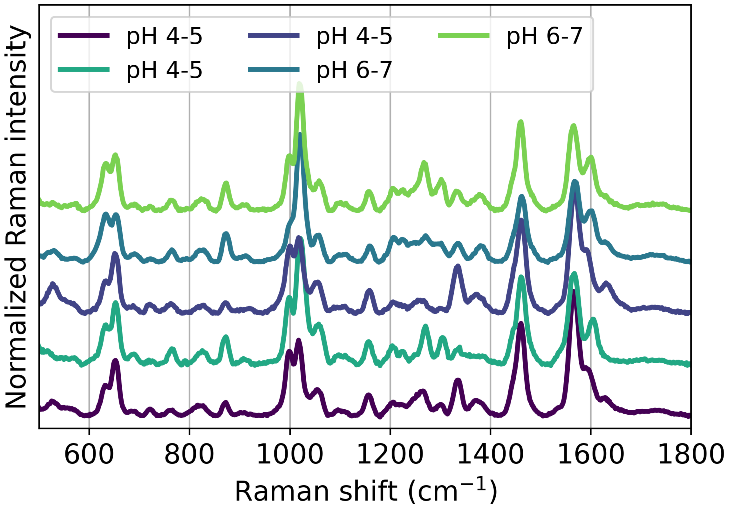

Figure S6: Raman spectra of solid state $[(N_4Py)Fe(II)(OH_2)]^{2+}$ prepared by drop coat deposition. Conditions: $[(N_4Py)Fe(II)(CH_3CN)](BF_4)_2$ dissolved in H_2O at pH 6-7 or pH 4-5 (adjusted by HNO_3), drop cast on aluminum foil after which the H_2O was evaporated. Raman spectrum recorded by accumulation of five 10 s acquisitions at λ_{exc} 785 nm.

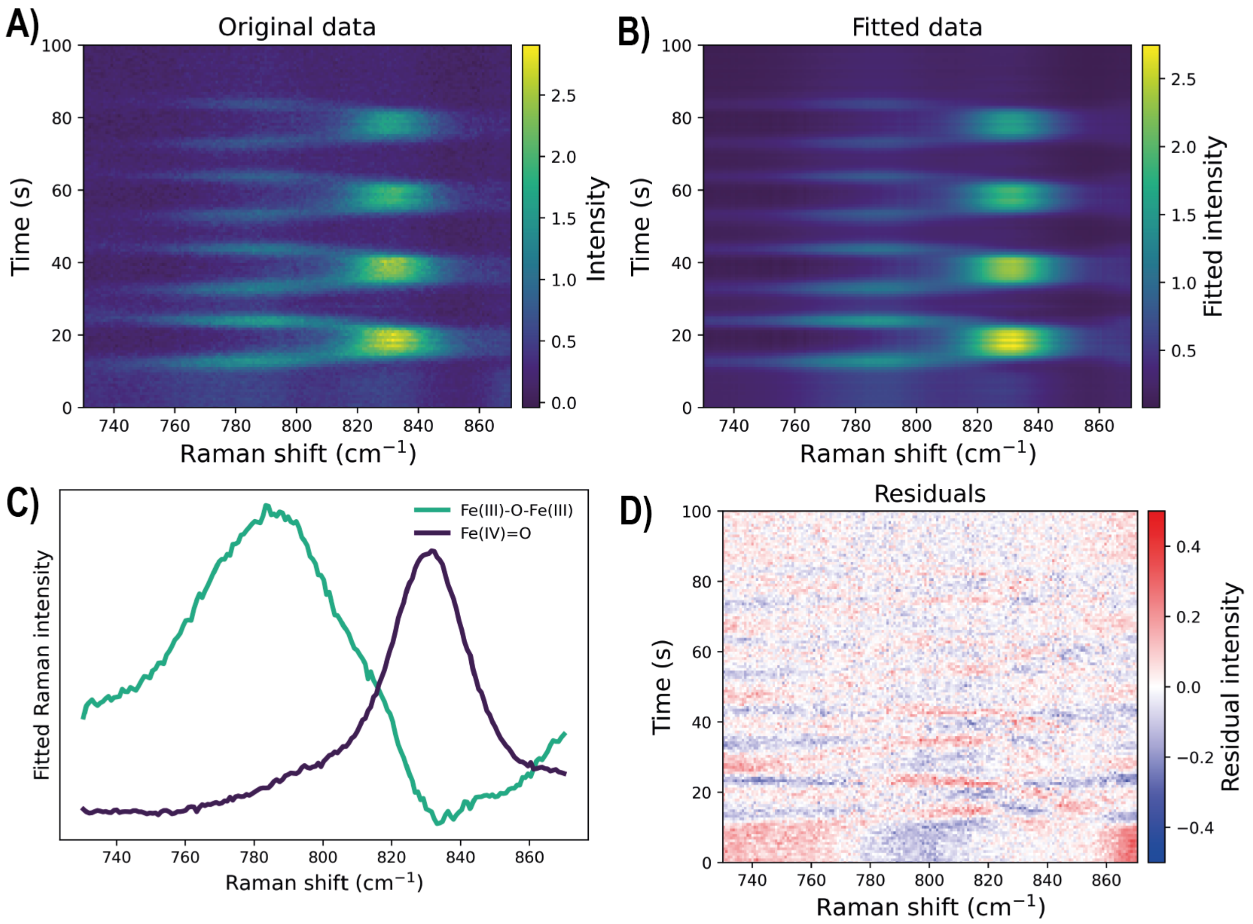

Figure S7: Multivariate curve resolution (MCR) analysis of SERS spectra from figure 4 between 730-870 cm^{-1} using two components to resolve the data. A) and B) depict the representation of the original data and fitted data, respectively. C) Fitted SERS intensities of the Fe(III)-OR and the Fe(IV)=O bands. D) The residual absorbance (actual absorbance minus fitted absorbance).

Multivariate Curve Resolution analysis of SERS spectra. Multivariate curve resolution (MCR) was conducted in Python using the pymcr package[8] in combination with non-negative least squares (NNLS) regression and a constraint that after each iteration the sum of the components involved for each data point sums-to-one (ConstraintNonneg, ConstraintNorm) (figure S8). These restrictions are reasonable for spectroscopic usage as long as all species involved have non-zero contribution to the data set.

The MCR analysis resulted in two component spectra and how these component spectra change over time with respect to each other (from now on called 'traces over time'). Note that these component spectra are not actual spectra of iron species, but merely mathematical shapes that describe the SERS data set. These component spectra do resemble the iron species, as indicated in the figure S7C. The fitted data set was reconstructed for comparison to the original SERS data set (figure S7A). This was done by multiplying the SERS intensity of a component spectrum at a certain wavenumber by the trace of this component at a certain time point, and the resulting values for each of the two components were summed. This was done for each wavenumber and each time point. This process can also be described as a matrix multiplication of the component spectra by the traces over time. The outcome of this calculation is the reconstructed data set and is represented in a color mesh (figure S7B). The difference between this reconstructed data set and the original data set is represented in the residuals color mesh figure S7D.

```python
# Import packages
import sys
import logging
from pymcr.constraints import ConstraintNonneg, ConstraintNorm
from pymcr.mcr import McrAR
from pymcr.regressors import NNLS

# Apply singular value decomposition as an initial guess for MCR.
u, sing, a = np.linalg.svd(dat) # apply the SVD

"""
This cell starts the MCR and takes parameters:
N_comp: number of components you want to used for fitting
u_vectors, a_vectores: The guesses made by the cell above
# NNLS (non negative least squares) is used for MCR.
# Hence, neither spectra nor concentrations can be negative (which makes sense for UV/vis).
"""
mcrar = McrAR(c_regr=NNLS(), st_regr=NNLS(), c_constraints=[], st_constraints=[])
def start_MCR(N_comp, u_vectors, a_vectors):

    logger = logging.getLogger('pymcr')
    logger.setLevel(logging.DEBUG)
    stdout_handler = logging.StreamHandler(stream=sys.stdout)
    stdout_format = logging.Formatter('%(message)s')  # Just a basic message akin to print statements
    stdout_handler.setFormatter(stdout_format)
    logger.addHandler(stdout_handler)
    mcrar.tol_increase = 50
    mcrar.tol_n_increase = 5000
    mcrar.tol_n_above_min = 5000
    mcrar.max_iter = 10000
    mcrar.tol_err_change  = 1E-12

    guess_spectra = []

    for i in range(N_comp):
        u_guess = u.T[i]
        a_complement = a[i]

        # This is done because the actual values of spectra and their
        # complements don't matter, rather their absolute values do.
        # If a spectrum is 'inversed' (the sum of all points is negative)
        # its importance can still be represented accurately by having a
        # negative complement.
        # While this works mathematically it does not make physical sense,
        # so we inverse them.
        if np.sum(u_guess) < 0:
            if np.sum(a_complement) < 0:
                u_guess = - u_guess # flipping when both u and v are mostly negative

        guess_spectra.append(u_guess)

    return mcrar.fit(dat.T, ST=guess_spectra)

# Start the MCR analysis from the initial guess from SVD and the chosen number of components.
start_MCR(4, u, a)
# The 'good' exiting message is "Change in err below tol_err_change (9.8861e-06). Exiting."
```

Figure S8: Python script for multivariate curve resolution (MCR).

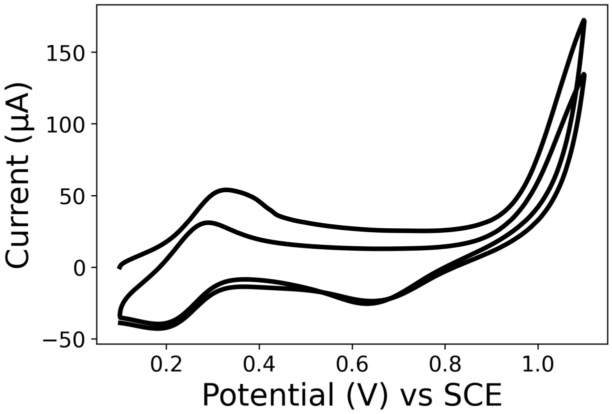

Figure S9: Cyclic voltammetry recorded concurrently with the SERS measurement of $[(N_4Py)Fe(II)(CH_3CN)]^{2+}$ in H_2O. Conditions: $[(N_4Py)Fe(II)(CH_3CN)](OTf)_2$ (2 mM), 0.1 M KNO_3 in H_2O, WE: gold bead, RE: Ag/AgCl, CE: Pt, scan rate: 0.1 V s^{-1}, SERS spectra recorded with 0.5 s acquisitions at λ_{exc} 785 nm.

Discussion relation Nernst equation and SERS intensities

The measured SERS intensity is proportional to the concentration of the species from which it arises by:[9]

$$I_{Raman} \propto [C] I_{laser} \alpha E_f \tag{1}$$

where I_{Raman} is Raman intensity, $[C]$ the concentration of the analyte, I_{laser} the intensity of the laser, α the polarizability of the analyte and E_f the surface enhancement factor. Therefore, the relative concentrations of the Fe(III) and Fe(IV)=O species is proportional to the relative intensity of the Fe(IV)=O and Fe(III)-O stretching bands as:

$$\frac{[Fe(IV)]}{[Fe(III)]} = \frac{I_{Fe(IV)=O}\ \alpha_{Fe(III)} E_{f,Fe(III)}}{I_{Fe(III)-O}\ \alpha_{Fe(IV)} E_{f,Fe(IV)}} \tag{2}$$

The laser intensity (I_{laser}) is constant and therefore cancels out in the relation. The polarizability (α) and the surface enhancement factor (E_f) of the two iron-species were estimated based on the assumption that the [Fe] at the maximum SERS intensity for Fe(III) and Fe(IV)=O are roughly equal, as at this point all of the iron complex within the region in which surface enhancement occurs (few nm from the electrode) is either present at Fe(III)-O

or Fe(IV)=O state. Therefore equation 2 may be used to estimate the ratio of the values of α and E_f for Fe(III) and Fe(IV)=O as:

$$\frac{[Fe(IV)]}{[Fe(III)]} = \frac{I_{Raman,max,Fe(IV)}}{I_{Raman,max,Fe(III)}} * \frac{\alpha_{Fe(III)} E_{f,Fe(III)}}{\alpha_{Fe(IV)} E_{f,Fe(IV)}} = 1 \tag{3}$$

At maximum SERS intensity (for Fe(III) at 0.6 V and Fe(IV)=O at >0.8 V, see figure 5C) the SERS intensity of Fe(IV)=O is ca. 2.3 times greater than Fe(III)-O, and hence, equation 3 is written as:

$$2.3 * \frac{\alpha_{Fe(III)} E_{f,Fe(III)}}{\alpha_{Fe(IV)} E_{f,Fe(IV)}} = 1 \tag{4}$$

And thus:

$$\frac{\alpha_{Fe(III)} E_{f,Fe(III)}}{\alpha_{Fe(IV)} E_{f,Fe(IV)}} = 0.42 \tag{5}$$

Therefore, equation 2 can be written as:

$$\frac{[Fe(IV)]}{[Fe(III)]} = \frac{I_{Raman,Fe(IV)} \alpha_{Fe(III)} E_{f,Fe(III)}}{I_{Raman,Fe(III)} \alpha_{Fe(IV)} E_{f,Fe(IV)}} = \frac{I_{Raman,max,Fe(IV)}}{I_{Raman,max,Fe(III)}} * 0.42 \tag{6}$$

Hence, we can relate the concentration ratios directly to the Raman intensity ratios of the two iron species. This calculation assumes that the magnitude of the surface enhancement is independent of electrode potential in this range, which is indeed unlikely to be the case, with a drop off in enhancement as the electrode is polarized positively. This will affect primarily the intensity of the Fe(IV)=O band. The Nernst equation relates the ratio of concentrations at the electrode to overpotential ($\eta = E - E^0(Fe^{III}/Fe^{IV})$) :

$$exp\left(-\frac{nF\eta}{RT}\right) = \frac{[Fe(IV)]}{[Fe(III)]} = \frac{I_{Raman,Fe(IV)} \alpha_{Fe(III)} E_{f,Fe(III)}}{I_{Raman,Fe(III)} \alpha_{Fe(IV)} E_{f,Fe(IV)}} \tag{7}$$

$$\left(\frac{\alpha_{Fe(IV)} E_{f,Fe(IV)}}{\alpha_{Fe(III)} E_{f,Fe(III)}}\right) exp\left(-\frac{nF\eta}{RT}\right) = \frac{I_{Raman,Fe(IV)}}{I_{Raman,Fe(III)}} \tag{8}$$

where R the ideal gas constant, T the temperature in kelvin, n number of electrons transferred in the reaction (1), and F the Faraday constant. The ratio however, is the con-

centration of the complexes at the electrode surface and the concentration will change as the distance from the electrode increases. Indeed the distance from the surface at which molecules can experience enhancement to the Raman scattering is several nanometers and hence the concentration of each species will lag behind that expected from the Nernst equation. This can be seen in the hysteresis observed in the plots shown in Figure 5c.

Fitting of the ratio of intensities of the Raman bands with respect to potential yields a best fit at 0.78 V (for the forward scan, figure S10) with the equation:

$$y = exp(\eta * C) \tag{9}$$

Variation of the value of C is not consistent with it being a simple constant (i.e. -F/RT) and hence manifests the concentration gradient with the distance from the electrode over which enhancement is observed.

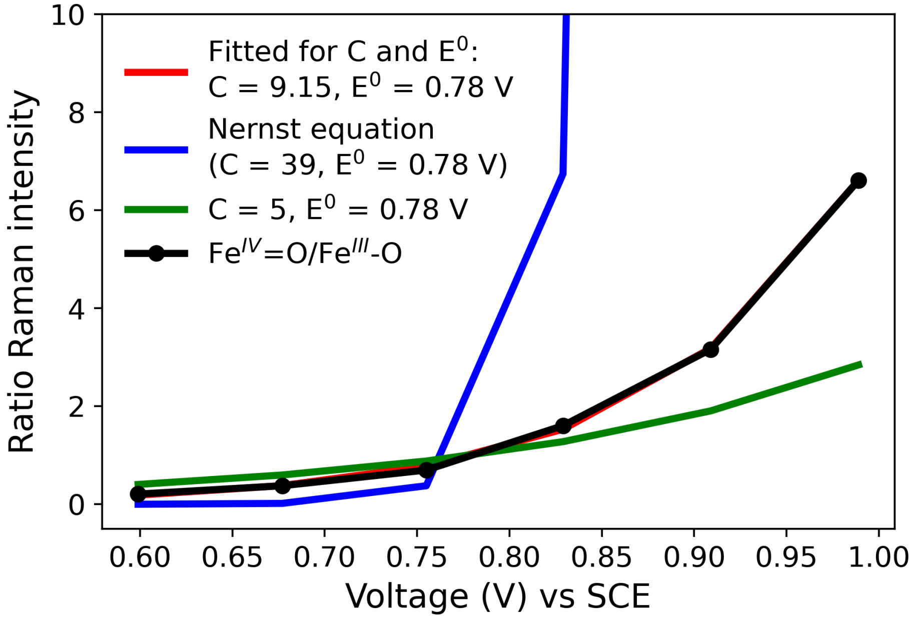

Figure S10: Plots of the ratio of intensities of the Fe(IV)=O and Fe(III)-OR stretching Raman bands from figure 4 vs. potential (black), fitted experimental data using equation 9 (red), Nernst equation using the fitted value of E^0 (blue), and equation 9 for C=5 and the fitted value of E^0 (green).

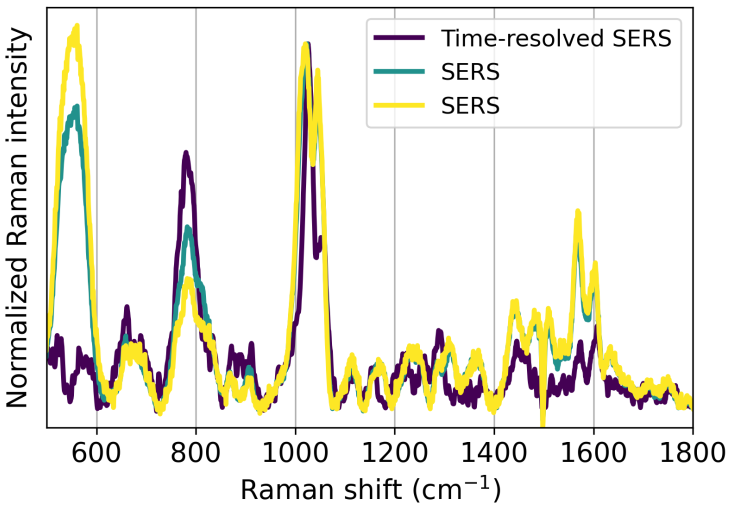

Figure S11: Comparison of time-resolved SERS spectrum at 0.5 V vs SCE recorded during cyclic voltammetry (0.5 s acquisition) and SERS spectra where the potential was held at 0.5 V vs SCE for a longer time (5 s, 5 accumulations, hence for approx. 25 s in total). Conditions: $[(N_4Py)Fe(II)(CH_3CN)](OTf)_2$ (2 mM) in 0.1 M KNO_3 in H_2O.

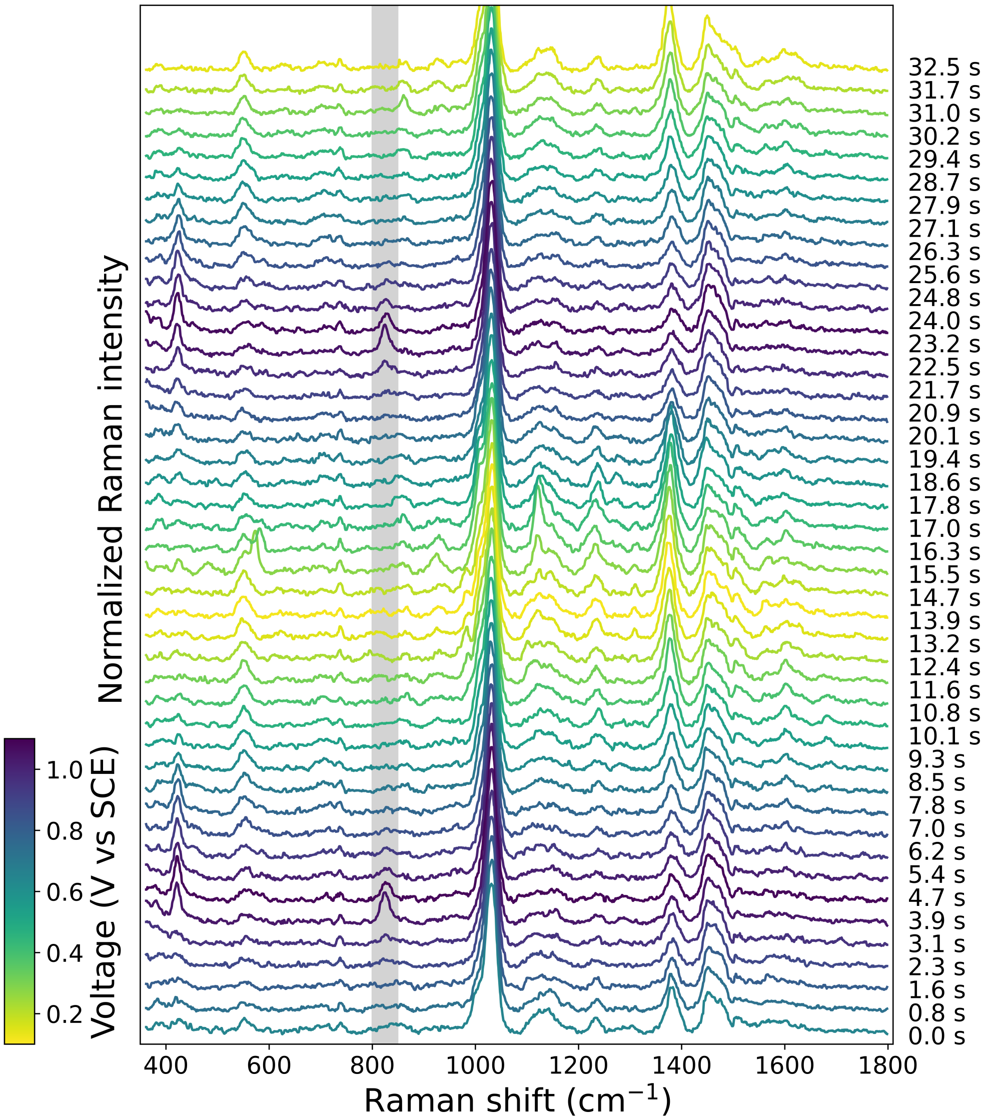

Figure S12: SERS spectra of $[(N_4Py)Fe(II)(HOMe)]^{2+}$ recorded during cyclic voltammetry in methanol. The potential and time is indicated by color (0.1 V - 1.1 V vs SCE) and indicated on the left and right, respectively. Conditions: $[(N_4Py)Fe(II)(CH_3CN)](OTf)_2$ (2 mM), 0.1 M KPF_6 in methanol, WE: gold bead, RE: Ag/AgCl, CE: Pt, scan rate: 0.1 V s^{-1}, SERS spectra recorded with 0.5 s acquisition time at 785 nm. Spectra were normalized to the methanol band at 1032 cm^{-1}.

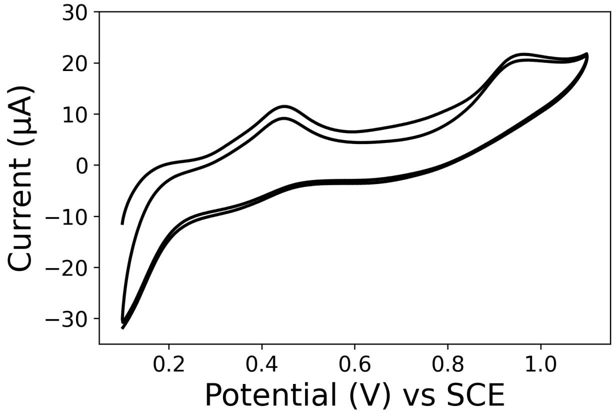

Figure S13: Cyclic voltammetry recorded concurrently with the SERS measurement of $[(N_4Py)Fe(II)(CH_3CN)]^{2+}$ in methanol. Conditions: $[(N_4Py)Fe(II)(CH_3CN)]OTf)_2$ (2 mM), 0.1 M KPF_6 in methanol, WE: gold bead, RE: Ag/AgCl, CE: Pt, scan rate: 0.1 V s^{-1}.

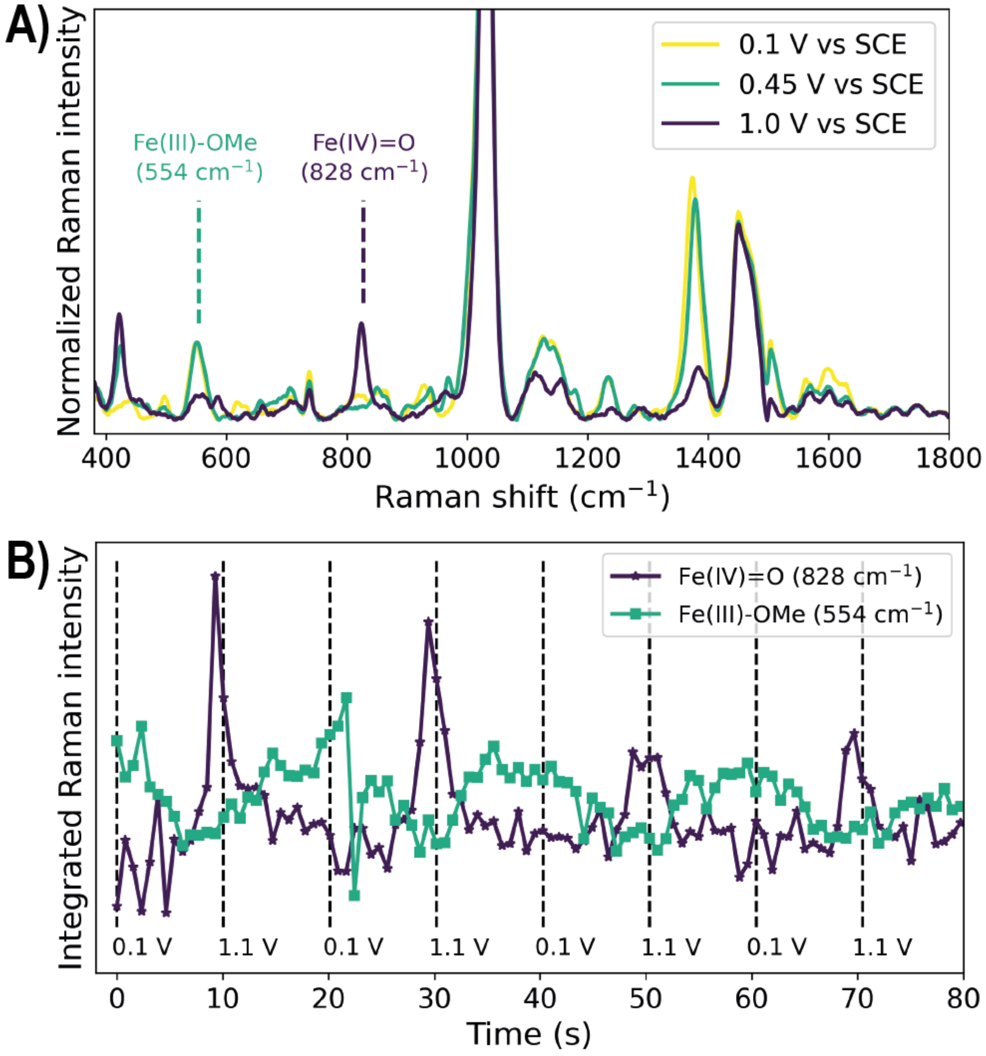

Figure S14: A) SERS spectra of $[(N_4Py)Fe(II)(HOMe)]^{2+}$ in methanol recorded with the electrode held at the potentials indicated; B) time plotted versus the integrated Raman intensities of Fe(IV)=O and Fe(III)-OMe. The minimum (0.1 V) and maximum (1.1 V) potentials during the cyclic voltammetry experiment are indicated by dotted black lines. $[(N_4Py)Fe(II)(CH_3CN)](OTf)_2$ (2 mM) in 0.1 M KPF_6 in methanol. SERS spectra recorded by accumulation of five 5 s acquisitions at 785 nm. Scan rate 0.1 V s^{-1}, WE: gold bead, RE: Ag/AgCl, and CE: Pt. Spectra were normalized to the methanol band at 1032 cm^{-1}. Note: An AuO band is not present at 570 cm^{-1} here, as AuO formation requires H_2O which is not present in significant amounts in methanol, and hence the overpotential for its formation is more positive. Cyclic voltammograms of a roughened Au bead in MeOH and H_2O are shown in (figure S17).

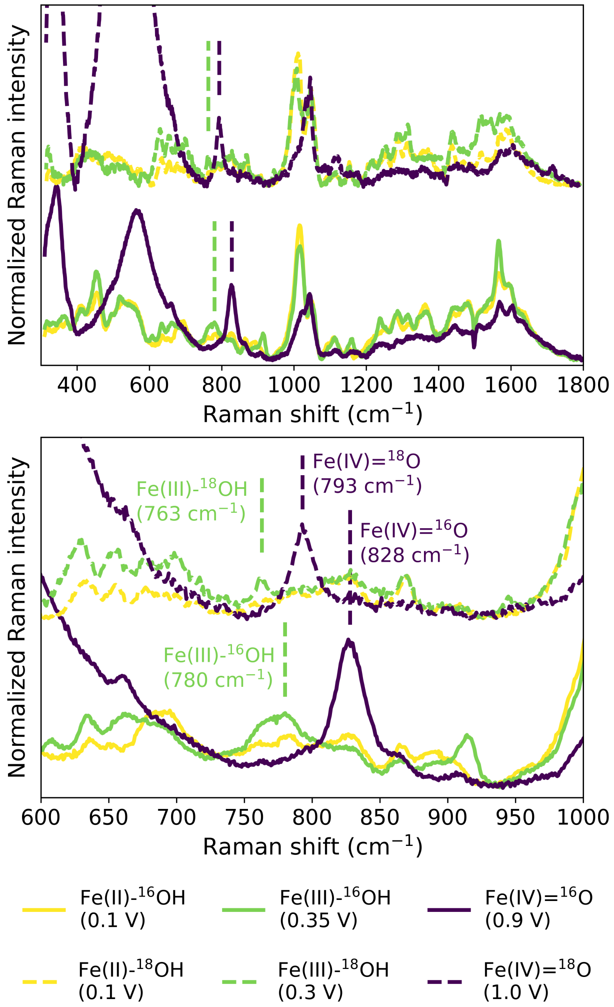

Figure S15: Surface enhanced Raman spectra of $[(N_4Py)Fe(II)(OH_2)]^{2+}$ in $H_2{}^{16}O$ (solid spectra) versus $H_2{}^{18}O$ (dashed spectra) recorded at the indicated potentials (vs SCE). (top) Full range between 300-1800 cm^{-1} and (bottom) expansion between 600-1000 cm^{-1}. Conditions: 2 mM $[(N_4Py)Fe(II)(CH_3CN)](OTf)_2$, 0.1 M KNO_3 in $H_2{}^{16}O$ or $H_2{}^{18}O$, roughened gold bead working electrode, Ag/AgCl reference electrode, and Pt counter electrode. Spectra were recorded with five times 5 s acquisitions at λ_{exc} 785 nm.

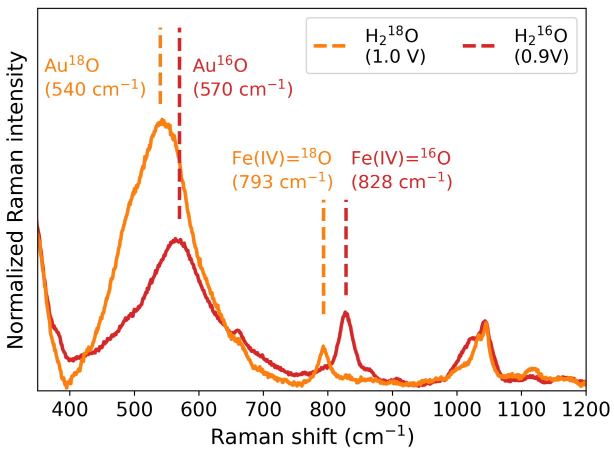

Figure S16: SERS spectra of $[(N_4Py)Fe(IV)=O]^{2+}$ in H^{16}_2O (red) and H^{18}_2O (orange) recorded at 0.9 V and 1.0 V vs SCE, respectively. Conditions: 2 mM $[(N_4Py)Fe(II)(CH_3CN)](OTf)_2$, 0.1 M KNO_3 in H^{16}_2O or H^{18}_2O, roughened gold bead working electrode, Ag/AgCl reference electrode, and Pt counter electrode. Spectra were recorded with five times 5 s acquisitions at λ_{exc} 785 nm.

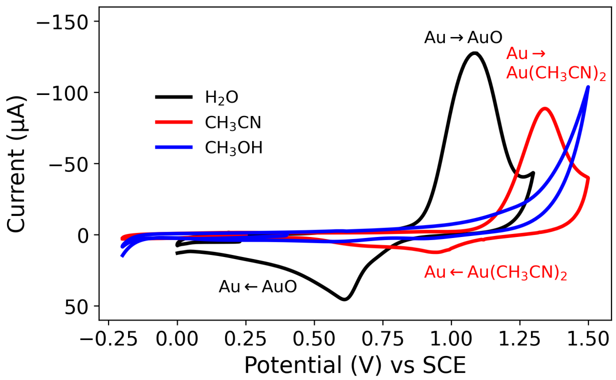

Figure S17: Cyclic voltammetry at a roughened gold bead in H_2O (pH 6 adjusted by HNO_3, 0.1 M KNO_3, black), in methanol (0.05 M KPF_6, blue), and in acetonitrile (0.1 M $TBAPF_6$, red) vs SCE, respectively. Roughened gold bead working electrode, SCE reference electrode, and Pt counter electrode.

References

(1) Collins, M. J.; Ray, K.; Que, L. Electrochemical Generation of a Nonheme Oxoiron(IV) Complex. *Inorg. Chem.* 2006, **45**, 8009–8011.

(2) Lee, Y.-M.; Kotani, H.; Suenobu, T.; Nam, W.; Fukuzumi, S. Fundamental Electron-Transfer Properties of Non-heme Oxoiron(IV) Complexes. *J. Am. Chem. Soc.* 2008, **130**, 434–435.

(3) Wang, D.; Zhang, M.; Bühlmann, P.; Que, L. Redox Potential and C-H Bond Cleaving Properties of a Nonheme Fe =O Complex in Aqueous Solution. *J. Am. Chem. Soc.* 2010, **132**, 7638–7644.

(4) Wang, D.; Ray, K.; Collins, M. J.; Farquhar, E. R.; Frisch, J. R.; Gómez, L.; Jackson, T. A.; Kerscher, M.; Waleska, A.; Comba, P.; Costas, M.; Que, L. Nonheme Oxoiron(IV) Complexes of Pentadentate N5 Ligands: Spectroscopy, Electrochemistry, and Oxidative Reactivity. *Chem. Sci.* 2013, **4**, 282–291.

(5) Nishida, Y.; Morimoto, Y.; Lee, Y.-M.; Nam, W.; Fukuzumi, S. Effects of Proton Acceptors on Formation of a Non-Heme Iron(IV)–Oxo Complex via Proton-Coupled Electron Transfer. *Inorg. Chem.* 2013, **52**, 3094–3101.

(6) Fukuzumi, S. Electron-Transfer Properties of High-Valent Metal-Oxo Complexes. *Coord. Chem. Rev.* 2013, **257**, 1564–1575.

(7) Draksharapu, A.; Rasheed, W.; Klein, J. E. M. N.; Que, L. Facile and Reversible Formation of Iron(III)–Oxo–Cerium(IV) Adducts from Nonheme Oxoiron(IV) Complexes and Cerium(III). *Angew. Chem. IE* 2017, **56**, 9091–9095.

(8) Camp, C. H. pyMCR: A Python Library for Multivariate Curve Resolution Analysis with Alternating Regression (MCR-AR). *J. Res. Natl Inst. Stand. Tech.* 2019, **124**, 124018.

(9) McCreery, R. Raman Spectroscopy for Chemical Analysis; Wiley, 2000
